# Peering into the black box: a meta-analysis of how clinicians use decision aids during clinical encounters

**DOI:** 10.1186/1748-5908-9-26

**Published:** 2014-02-22

**Authors:** Kirk D Wyatt, Megan E Branda, Ryan T Anderson, Laurie J Pencille, Victor M Montori, Erik P Hess, Henry H Ting, Annie LeBlanc

**Affiliations:** 1 Mayo Medical School, Mayo Clinic College of Medicine, 200 First St. SW, 55905 Rochester, MN, USA; 2 Knowledge and Evaluation Research Unit, Mayo Clinic, 200 First St. SW, Rochester, MN, USA; 3 Center for the Science of Healthcare Delivery, Mayo Clinic, 200 First St. SW, Rochester, MN, USA; 4 Department of Health Sciences Research, Division of Biomedical Statistics and Informatics, Mayo Clinic, 200 First St. SW, Rochester, MN, USA; 5 Department of Emergency Medicine, Mayo Clinic, 200 First St. SW, Rochester, MN, USA; 6 Department of Medicine, Division of Endocrinology, Mayo Clinic, 200 First St. SW, Rochester, MN, USA; 7 Department of Cardiology, Mayo Clinic, 200 First St. SW, Rochester, MN, USA; 8 Department of Health Sciences Research, Division of Health Care Policy and Research, Mayo Clinic, 200 First St. SW, 55905 Rochester, MN, USA

**Keywords:** Shared decision making, Decision aids, Meta-analysis, Fidelity, Patient involvement, Patient-centered care

## Abstract

**Objective:**

To quantify the extent to which clinicians use clinically-efficacious decision aids as intended during implementation in practice and how fidelity to usage instructions correlates with shared decision making (SDM) outcomes.

**Methods:**

Participant-level meta-analysis including six practice-based randomized controlled trials of SDM in various clinical settings encompassing a range of decisions.

**Results:**

Of 339 encounters in the SDM intervention arm of the trials, 229 were video recorded and available for analysis. The mean proportion of fidelity items observed in each encounter was 58.4% (SD = 23.2). The proportion of fidelity items observed was significantly associated with patient knowledge (p = 0.01) and clinician involvement of the patient in decision making (p <0.0001), while no association was found with patient decisional conflict or satisfaction with the encounter.

**Conclusion:**

Clinicians’ fidelity to usage instructions of point-of-care decision aids in randomized trials was suboptimal during their initial implementation in practice, which may have underestimated the potential efficacy of decision aids when used as intended.

## Introduction

Shared decision making (SDM) is a process whereby patients and clinicians deliberate together to make decisions that reflect the best available evidence about the existing options synthesized with patients’ preferences, values, goals and context [1]. While many definitions of SDM exist, themes found most consistently in these definitions include incorporation of patient preferences and values, presentation of options to the patient, partnering with the patient, facilitating patient participation, educating the patient, and presentation of benefits and risks [2]. A substantial body of evidence indicates that use of decision aids improve outcomes thought to be important components of SDM, including patient knowledge, decisional conflict (especially as it relates to being unclear about personal values and feeling uninformed), and accuracy of patient risk perception when compared to usual care. The tools also increase the extent to which clinicians engage patients in the decision making process during consultations [3].

While many decision aids have been designed for patient use outside of the clinical encounter [3], our research group has designed and studied decision aids for use during the clinical consultation as tools to support clinicians’ efforts to engage patients in SDM [4-8]. These decision aids are designed to create conversations about the available options and support those conversations with evidence-based information about the relevant pros and cons associated with each option presented, while taking into consideration clinicians’ expertise and patients’ personal values and preferences [9,10]. Our user-centered design approach makes it more likely that the decision aid will meet the needs of both users (i.e., clinicians and patients) and accommodate the pressures of time-limited encounters. To date, our SDM trials have demonstrated that our decision aids have been able to create meaningful conversations, increase patient knowledge relevant to the choice to be made and increase the extent to which clinicians involve patients in the decision making process while having variable impact on choice and adherence to choice [5-7,11]. The efficacy of our decision aids is based on the assumption that clinicians use them in accordance with the principles of SDM, which we communicate through simple and brief usage instructions.

Qualitative analysis of video recordings from our first trial, ‘Statin Choice,’ clearly suggested, however, that while most clinicians appear to use the decision aid as intended, its design accommodated a range of clinician uses, some of which no longer resembled SDM [11,12]. For instance, clinicians would use the decision aid to justify their personal biases, as opposed to exploring the patient’s preferences. Video recordings continued across all of our trials, in both intervention and usual care arms, creating a rich and unique database to evaluate the extent and manner in which clinicians used decision aids as intended during clinical encounters and how fidelity to intended use of decision aids modifies their efficacy.

We reviewed video recordings from the SDM intervention arm of trials where decision aids were used at the point-of-care by clinicians and patients, and we extracted measures of fidelity to decision aid usage instructions, patient decisional conflict, knowledge, involvement in the decision making process, and satisfaction with the encounter. Our objective was to use these data to quantify the extent to which clinicians follow the usage instructions of the decision aids and understand how following these instructions affects important SDM outcomes, such as patient knowledge, patient decisional conflict, and clinician engagement of the patient. Our hypothesis was that fidelity to decision aid usage instructions was less than perfect and resulted in suboptimal SDM outcomes measured in the included trials.

## Methods

### Study design

We conducted a participant-level meta-analysis to assess the extent to which clinicians used decision aids as intended with their patients during clinical encounters in practice-based, randomized, controlled trials of decision aids. The Mayo Clinic Institutional Review Board (IRB) approved the study procedures described herein. Moreover, for each of the trials included in this study, clinicians and patients provided written informed consent for all study procedures, including video recording.

### Decision aid development

The decision aids involved in our trials were designed with extensive input from patients and clinicians to fit the context of their intended use, the individual clinical encounter, and the fast-paced setting of most practices [9]. Our decision aids were designed to be brief, to the point, and user-oriented, so that they would be easy to use and adaptable to the clinical scenario. The purpose of the decision aids was to serve as a guide that presents the best available evidence, utilizes the clinician’s expertise, and elicits the patient’s preferences and values with the intent of creating a conversation in the context of SDM. The training that accompanied these decision aids was minimal and included brief video clips and storyboards that demonstrate basic use of the targeted decision aid (publicly available at http://shareddecisions.mayoclinic.org). We intended for this training to be minimal in order to facilitate the easy adoption and implementation of our decision aids both during and after the trials. On-site study coordinators were available to do one-on-one demonstrations on an as-needed basis during the trials.

### Data source

We included all but one completed SDM trial conducted through the Wiser Choices Program of the Knowledge and Evaluation Research (KER) Unit at Mayo Clinic in Rochester, MN, USA (Table 1). All included trials were practice-based, 2-arm, randomized controlled trials enrolling clinicians and patients at the point of care in Southeastern Minnesota, USA. They included a similar SDM intervention (i.e., a brief decision aid to be used by clinicians and patients during clinical encounters) and assessed similar measures of SDM processes and outcomes [7,13-15]. These trials also measured the comparative impact on clinical and utilization outcomes. We excluded the ‘Statin Choice’ trial from this study as the overall design and structure of the study were different from the others [11].

**Table 1:** Wiser choices clinical trials included in the study

	**AMI choice**	**Chest pain choice**	**Decision aids for diabetes (DAD)**	**Diabetes choice**	**Osteoporosis choice I/II**^**a**^
**Trial registration**	NCT00888537	NCT01077037	NCT01029288	NCT00388050	NCT00578981, NCT00949611
**Decision**	Intensive medical treatment to reduce 6-month mortality risk after acute myocardial infarction (AMI)	Disposition after ruling out acute coronary syndrome for patients presenting with chest pain	Use of statins to reduce 10-year cardiovascular risk (Statin choice) and choice of antihyper-glycemic medications (Diabetes choice)	Choice of antihyper-glycemic medications	Use of bisphosphonate to reduce 10-year fracture risk among post-menopausal women
**Setting**	Hospital	Emergency department	Primary care practices	Primary care practices	Primary care practices

^a^Osteoporosis I and II trials were pooled together as one trial is the continuity of the other.

### Data extracted

We used all available video recordings of clinical encounters and extracted measures from baseline, post-encounter and follow-up surveys from patients and clinicians, pharmacy records, and third-observer reports. We included all available data from the intervention arm of each trial.

### Fidelity (intended use of the decision aid)

Considering the minimal training clinicians received in the use of the decision aids and their varying knowledge and application of SDM principles, we developed a fidelity checklist for each of the decision aids to ensure that clinicians were using the decision aids as intended (see Additional file 1). The scale was developed by the developers of the decision aids to reflect the obligatory elements that should be part of the conversation generated by using the decision aid. Given that each tool is unique, the fidelity scale differs for each decision aid. The fidelity checklist for each decision aid comprised a different number of items (all on a present/absent scale); thus total scores are reported as a percentage of items (i.e., behaviors) present. Some items on the checklist are overlapping, as they must occur in a stepwise fashion (e.g., Did the clinician describe the risk as a natural frequency? Did the clinician describe the time horizon for the risk? Did the clinician describe the risk graphically? Did the clinician describe the risk reduction as a natural frequency? Did the clinician describe the risk reduction graphically?). For the present study, each of these questions was scored independently. Additional items captured data for descriptive purposes and were not included in calculation of the fidelity score. Additional file 1 details which items were included in calculating the fidelity score. The fidelity score is calculated as a percentage of items performed by the clinician. We considered, based on clinical opinion, a fidelity threshold of 66% (2/3 of items addressed) as a successful (i.e., ‘as intended’) use of the decision aids. A perfect fidelity score (i.e., 100%) would involve the clinician incorporating all of the components of the decision aid training. While we hypothesize that higher fidelity scores are optimal, we recognize that variability and imperfect scores may result as clinicians tailor the decision aid to time-limited encounters or to patients with whom they have already previously discussed components included on the decision aid. Taking into account this variability, we chose a 66% fidelity score as the threshold for ‘acceptable’ use, recognizing that this threshold is somewhat arbitrary. Examples of unskilled uses that could result in low fidelity score include using the decision aid to convince a patient of the clinician’s personal bias without walking the patient through the decision aid, and omission of several key components of the decision aid deemed essential for patient understanding of their clinical context (e.g., sharing baseline risk as a percentage without explaining the percentage as a natural frequency, without explaining it visually, or without explaining the time course over which the risk applies).

The Decision Aids to Enhance Shared Decision Making for Diabetes (‘DAD’) trial video recordings were reviewed in duplicate with a percentage of overlap (20%) to ensure adequate reproducibility (concordance = 95%). The remainder of trials were reviewed by a third reviewer, with this reviewer’s reproducibility only for the ‘Chest Pain Choice’ trial verified by comparison with an additional reviewer and adequate reproducibility (concordance = 92%). Scores from only one reviewer per encounter were used to calculate the fidelity score.

While some video recordings included the entire clinical encounter, others included most but not all of the clinical encounter (e.g., the entire consultation with video recording stopped at the point of the physical examination, or the beginning of the consultation through the use of the decision aid), and some included only the component of the clinical encounter in which the decision aid was used. Because the entire clinical consultation was not consistently recorded and because the main outcome of interest was the manner in which the clinician did or did not implement the decision aid as instructed, only the portion of the clinical encounter in which the decision aid was used was analyzed with the fidelity scale.

### Outcome measures

Patients’ levels of decisional conflict were assessed immediately after consultations using the Decisional Conflict Scale (DCS) [16]. Specifically, the scale measures personal perception of uncertainty and explores the factors contributing to that uncertainty. The scale includes 5 subscales and 16 items on a 0 to 4 Likert scale, where scores can be reported globally or for each subscale individually. We reported the scores for each subscale, transposing them on a 0 to 100 range, with higher scores indicating greater comfort with decision making. The number of subscales assessed varied across trials; we assessed two of the five subscales (Information and Effectiveness) in the ‘AMI Choice’ trial, three of the five (Information, Effectiveness and Support) in the ‘DAD’ trial, and all five subscales in the remaining trials.

Patients answered true/false knowledge questions pertaining to information considered essential in the decision-making process for the clinical problem at hand, mainly around cognizance of the problem, its alternatives, and associated main benefits and risks. The total knowledge scores were expressed as a percent of the maximum possible score. When pertinent, we also asked patients to indicate their individual estimated risk of adverse outcomes (e.g., 10-year coronary risk), which we then compared with the actual calculated risk provided in the decision aid. Satisfaction with the encounter was measured using patients’ willingness to recommend the way they made the decision (i.e., use of the decision aid) to others on a 7-point Likert scale, converted into two categories: recommend (1 to 2) or not (5 to 7). Chart reviews provided evidence about the action patients took (i.e., actual decision), which we compared to their declared decision on post-visit questionnaires (i.e., intended decision).

The OPTION scale, a third-observer scale, was used to evaluate clinicians’ efforts to involve patients in SDM [17]. The scale has 12 items scored on a 0 to 4 scale, which are then summed to form the total score (maximum = 48). For ease of interpretation, we transposed this score on a 0 to 100 range, with higher scores indicating greater involvement in decision making.

### Socio demographic data

For each included trial, we extracted patients’ gender, age, education, income, marital status, and insurance plan, in addition to clinicians’ gender and type (i.e., attending physician, resident physician, nurse).

### Statistical analyses

We presented demographics and clinical characteristics as counts and percentages for categorical values and as means and standard deviations for continuous values. We conducted a sensitivity analysis using the Wilcoxon rank sum test for continuous and Fisher’s exact for categorical outcomes to test for differences in patient outcomes when the clinical encounter was recorded compared to when it was not. We are presenting each trial’s results individually and overall to report the results of the video-recorded patients from the decision aid arm of the trials that have not been presented elsewhere. For individual trial results, we calculated correlations among continuous outcomes and fidelity using the Spearman’s rank coefficient, while logistic regression was used for binary outcomes. We conducted meta-analysis of the included trials using a generalized linear mixed model that was stratified by trial for continuous outcomes. We modeled adherence to decision and the fidelity threshold by logistic regression, stratified by trial.

We conducted all analyses using SAS 9.2 (Cary, NC) and considered two-sided p-values <0.05 as significant. We recorded and managed study data using the Research Electronic Data Capture (REDCap) system [18].

## Results

Table 2 shows characteristics of the included trials, of the recording material available, and of the patient and clinician participants. We were able to collect 206 video and 23 audio recordings out of a possible 339 encounters (68%) from the SDM arms of the trials.

**Table 2:** Characteristics of included patients and clinicians

	**AMI choice**	**Chest pain choice**	**Decision aids for diabetes (DAD)**	**Diabetes choice**	**Osteoporosis choice I/II**^**a**^	**All trials**
Patients enrolled to the SDM intervention arm, n	53	101	53	48	84	339
Video recordings available, n (%)^b^	31 (58)	98 (97)	23 (43)	28 (58)	49 (58)	229 (68)
**Patients**						
N (% of female)	7 (23)	56 (57)	4 (17)	14 (50)	49 (100)	131 (57)
Age, mean (SD)	65 (7)	54 (12)	60 (11)	63(11)	67 (9)	60 (12)
K-12 education, n (%)^c^	12 (41)	27 (28)	9 (39)	10 (36)	16 (34)	74 (33)
Income <40 k/year, n (%)^d^	14 (47)	25 (28)	6 (26)	13 (50)	16 (38)	74 (35)
Married, n (%)^e^	20 (65)	45 (80)	18 (78)	12 (86)	28 (72)	123 (75)
Commercial insurance, n (%)^f^	16 (52)	75 (77)	8 (89)	12 (67)	17 (35)	128 (62)
**Clinicians**						
N (% female)	3 (100)	51 (25)	16 (31)	16 (44)	36 (47)	122 (37)
Encounters per clinician, mean, median (range)	10.3, 10 (5,16)	1.9, 2 (1, 6)	1.9, 1 (1, 8)	2.6, 2 (1, 8)	1.7, 1 (1,8)	2.2, 1 (1, 16)
Type^g^						
Staff physician, n (%)	~	20 (41)	12 (75)	13 (81)	25 (69)	70 (58)
Physician in training, n (%)	~	26 (53)	2 (12.5)	0 (0)	6 (17)	34 (28)
Nurse or nurse practitioner, n (%)	3 (100)	3 (6)	2 (12.5)	3 (19)	5 (14)	16 (14)

^a^Osteoporosis I and II trials were pooled together as one trial is the continuity of the other; ^b^Some recordings were not available due to technical issues (i.e., missing sound, not readable); ^c^Values missing for education (AMI = 2, Chest Pain Choice = 3, Osteoporosis choice I/II = 2); ^d^Value missing for income (AMI = 1, Chest Pain Choice = 10, Diabetes choice = 2, Osteoporosis choice I/II = 7); ^e^Values missing for marital status (Chest Pain Choice = 42, Diabetes = 14, Osteoporosis choice I/II = 10); ^f^Values missing for insurance (DAD = 14, Diabetes choice = 10); ^g^Values missing for clinician type (Chest Pain Choice = 2).

### Fidelity (intended use of the decision aid)

Across all trials, there were six encounters in which clinicians did not use the decision aid with their patients. High level of concordance was found between fidelity scores from the third reviewer and original reviewer pairs (concordance between 76% and 92%) for the ‘AMI Choice’, ‘Chest Pain Choice’, ‘Diabetes Medication Choice’, and ‘Osteoporosis Choice’ I/II trials [19]. Considering the high level of concordance, in addition to the fact that he trained and supervised the reviewers for the ‘DAD’ trial, the third reviewer did not assess the video recordings of the ‘DAD’ trial. All results used in this study, except for those of the ‘DAD’ trial, are from the third reviewer (KDW).

Across all recordings, we observed 58% (95% CI 56, 62) of the fidelity items in video recordings of encounters. The range of fidelity scores was 0% to 100%. Almost half (47%) of the encounters addressed at least 66% of the fidelity items. Trial-specific fidelity was on average lower (56%) and varied across trials (39-66%). Regarding the key expected behaviors, the degree of information sharing and facilitation of the decision making process was similar across trials, while elicitation of values and preferences was highly variable between trials (Table 3). For instance, clinicians elicited patients’ preferences and values in 89% of the encounters within the ‘Diabetes Medication Choice’ trial compared to none within the ‘AMI Choice’ trial (Table 3). Clinicians made recommendations in 46% of the encounters, and 75% of these recommendations were unsolicited.

**Table 3:** Fidelity scores

	**AMI choice**	**Chest pain choice**	**Decision aids for diabetes (DAD)**	**Diabetes medication choice**	**Osteoporosis choice**	**All**
Fidelity score^a^	61.0 (57.5, 64.5)	52.4 (47.1, 57.6)	62.8 (51.1, 74.6)	64.6 (56.3, 72.9)	63.8 (58.2, 69.3)	58.5 (55.5, 61.5)
Information component^a^	65.7 (57.3, 74.1)	52.4 (47.1, 57.6)	39.1 (23.8, 54.5)	54.6 (44.1, 65.0)	63.8 (58.2, 69.3)	55.6 (52.1, 59.0)
Decision making process components^a^	61.3 (56.7, 65.9)	63.3 (58.9, 67.6)	68.1 (58.4, 77.9)	62.1 (53.4, 70.9)	61.2 (54.6, 67.8)	62.9 (60.2, 65.7)
Eliciting values/preferences^b^	0 (0)	18 (18)	12 (52)	25 (89)	7 (14)	62 (27)
Fidelity grouping^b,c^	10 (32)	47 (48)	12 (52)	13 (46)	27 (55)	109 (47)

^a^Percentage of fidelity items addressed in the encounter; Mean (95% CI), unadjusted scores.

^b^Count of encounters with behavior/meeting criteria (%).

^c^66% or more of fidelity items exhibited within the encounter.

### Association of fidelity with trial outcomes

We found significant associations between fidelity scores and patient knowledge (p = 0.01) and patient involvement in the decision making process (OPTION score) (p <0.0001) (Table 4). We found no significant associations with the decisional conflict score or any of its subscales, patient knowledge of risk, patient satisfaction with the decision-making process, or concordance between the decision made and patient action (Table 4). There was no evidence of a fidelity threshold; encounters with ≥66% of fidelity items addressed did not differ in respect to outcomes. Patients’ concordance between the action that was taken and the decision that was made within the encounter was not significantly changed by a higher degree of fidelity to the items being examined (Table 5).

**Table 4:** **Fidelity association with SDM outcomes**^**a**^

	**AMI choice**	**Chest pain choice**	**Decision aids for diabetes (DAD)**	**Diabetes medication choice**	**Osteoporosis choice**	**All**^**c**^
Patient involvement (OPTION score)	0.563	0.006, R = 0.276	0.115	0.002, R = 0.556	0.641	<0.0001, R^2^ = 0.542
DCS overall	0.236	0.430	0.121	0.966	0.679	0.845, R^2^ = 0.023
DCS informed	0.146	0.216	0.174	0.593	0.646	0.589, R^2^ = 0.034
DCS effective	0.608	0.864	0.194	0.893	0.236	0.667, R^2^ = 0.031
DCS support	~	0.399	0.457	0.674	0.576	0.261, R^2^ = 0.047
DCS values	~	0.683	~	0.861	0.927	0.821, R^2^ = 0.013
DCS certainty	~	0.946	~	0.968	0.470	0.690, R^2^ = 0.018
Knowledge	~	0.065	0.194	0.053, R = 0.369	0.001, R = 0.455	0.011, R^2^ = 0.095
Knowledge of risk estimates^b^	0.341	0.639	~	~	0.857	0.861
Satisfaction (willingness to recommend to others)^b^	0.481	0.269	0.470	0.649	0.952	0.251

Abbreviations: *DCS* Decisional Conflict Scale; *OPTION* Observing Patient Involvement.

^a^P-value of Spearman’s rank correlation with R for significant values.

^b^P-value for logistic model results; overall model stratified by study.

^c^Generalized linear mixed model adjusted by fixed effect of fidelity and random effect of study.

**Table 5:** Adherence to decision values and association with fidelity

	**AMI choice**	**Chest pain choice**	**DAD**	**Diabetes medication choice**	**Osteoporosis choice**	**All**^**a**^
**Adherent to decision, n (%)**	23 (88)	66 (70)	11 (85)	20 (83)	19 (63)	139 (74)
Mean difference of fidelity for Adherent vs. not, % (95% CI)	−2 (−15, 11)	2 (−9, 14)	−6 (−46, 33)	10 (−13, 33)	−13 (−29, 3)	0.85 (0.2, 3.6)

^a^Odds ratio (95% CI), logistic regression stratified by study.

We conducted a sensitivity analysis using a generalized linear model for continuous and a logistic regression model for categorical outcomes to test for differences in patient outcomes when the clinical encounter was recorded compared to when it was not, adjusting by treatment arm. Differences were found among outcomes for knowledge of risk (AMI Choice, p = 0.003), DCS certainty (Chest Pain Choice, p = 0.007), patient knowledge (Osteoporosis Choice, p = 0.005) and satisfaction (Diabetes Medication Choice, p = 0.01; Additional file 2).

### Fidelity with repeated use of decision aids

We assessed whether fidelity scores increased with repeated use of decision aids. A total of 47 clinicians used decision aids more than once, 25 used them more than twice, and 15 used them more than 3 times, and we found that fidelity scores increased for 29% of clinicians, stayed the same in 40%, and decreased in 31%. The threshold of at least 66% of fidelity items addressed in the first encounter appeared to suggest a trend toward better results in the subsequent encounters for those with four or more encounters, but due to limited number of clinicians this could not be tested. Table 6 shows patient and clinician characteristics of encounters in which clinicians used decision aids at least thrice in the included trials.

**Table 6:** **Compliant (>66%) in fidelity items in the first three consecutive encounters for clinicians who participated in included trials at least three times**^**a**^

	**Compliant in all encounters**	**Not compliant in any encounters (n = 5)**	**Compliant in at least one but not all encounters (n = 12)**
	**(n = 8)**		
	**n (%)**	**n (%)**	**n (%)**
***Clinician characteristics***			
Type: Staff physician	5 (63)	3 (60)	7 (58)
Physician in training	3 (38)	1 (20)	3 (25)
Nurse or nurse practitioner	0	1 (20)	2 (17)
Female	3 (38)	1 (20)	4 (33)
***Patient characteristics from encounters***			
Sex: Majority male	4 (50)	2 (40)	5 (42)
Majority female	4 (50)	3 (60)	7 (58)
Age, y: Majority <65	7 (87.5)	2 (40)	11 (92)
Majority ≥65	1 (12.5)	3 (60)	1 (8)
Education: majority college^b^	5 (25)	3 (60)	10 (83)
Majority HS or less	3 (25)	2 (40)	2 (17)

Abbreviations: *HS* High School.

^a^Clinicians with at least 3 encounters (n = 25) were included, the first three encounters were evaluated for compliance to fidelity of 66% or more in each encounter.

^b^College includes post-grad, four-year degree, two-year degree and vocational school.

## Discussion

We found, after the initial implementation effort, that clinicians used decision aids as intended only partially and inconsistently, that key elements of decision aid use were missing from most encounters, and that fidelity scores incompletely correlated with important outcomes of decision aid use. When decision aids were used, clinicians made recommendations in nearly one half of clinical encounters; moreover, three-fourths of these recommendations were unsolicited.

Interestingly, different components of the fidelity score were implemented variably between studies. The observation that clinicians did not elicit values/preferences during the ‘AMI Choice’ study was likely a consequence of the study design. ‘AMI Choice’ was the only included study where the clinician presenting the decision aid was not the patient’s clinician, and their only contact with the patient was for the exclusive purpose of delivering the decision aid intervention. In this sense, the decision aid was presented as more of an educational encounter.

### Limitations and strengths

While fidelity was correlated with OPTION score, it is possible that this observed association is spurious. While the intent of the fidelity scale was objective measurement of the mechanical steps of decision aid use by the clinician which are thought to facilitate SDM, the OPTION scale acted as a direct measure of the SDM process. Nonetheless, some overlap in scoring items is to be expected between different measures relating to SDM since the intended outcome being measured is similar. Thus, the finding of correlation between fidelity and patient involvement in the decision making process (i.e., OPTION scores) may in part be explained by the overlap in items between these measures: both evaluate the extent to which clinicians elaborated on the problem that requires a decision, operated within the context of uncertainty about the best course of action, presented more than one option to the patient, and explained the pros and cons associated with the options. However, the fidelity checklist did not include items similar to those on the OPTION scale addressing the extent to which clinicians explored patient expectations and concerns, verified patient understanding, provided opportunities to ask questions, assessed the patient’s preferred level of involvement, signaled the need for a decision making stage, or indicated the need to review the decision. Instead, the fidelity scale placed an emphasis on actions clinicians took to increase patient understanding of their options (e.g., ‘Did the clinician explain risk reduction graphically?’) and were often decision aid specific. Indeed, the significant relationship between fidelity and patient knowledge that we observed may reflect the emphasis that the fidelity checklist places on knowledge transfer behaviors as opposed to other behaviors thought to be associated with SDM. The observation that the fidelity checklist emphasized knowledge transfer behaviors over elicitation of patient preferences/values and partnership with patients yet still was associated with increased OPTION scores suggests that knowledge transfer serves to engage patients in shared decision making.

The recording of what takes place in the clinical consultation in the context of randomized trials of decision aids is an important component of understanding how these tools work. While logistically complicated for study personnel and participants, we managed to video record a fairly large proportion of encounters, but not all. Whether this introduces bias into our results is unclear; in our judgment, clinicians and patients that consent to video may consider themselves most compliant with study procedures and should be exhibiting the highest adherence to instructions of decision aid use. Thus, our findings may represent an overestimate of actual clinician fidelity.

Moreover, because the entire clinical consultation was not consistently recorded and our main outcome of interest was fidelity to the implementation of the decision aid, we analyzed only the portion of the clinical encounter where the decision aid was utilized in those recordings that were available. While we presume that this portion of the clinical consultation is what results in the measured outcomes (e.g., patient decisional conflict, patient knowledge), it is possible that other components of the clinical encounter that were not recorded or analyzed could have contributed to the outcomes of interest and that fidelity does not necessarily represent a direct correlation with SDM in all clinical encounters.

Beyond the ability to peer into the ‘black box’ of the clinical encounter, our study offers the strength of completing reproducible assessments while considering different decisions (e.g., medication options, disposition from the emergency department, risk reduction), clinical contexts (e.g., routine chronic care, emergency care, hospital discharge), patient types (return versus new) and clinician types (e.g., inpatient nurses, primary care physicians, emergency care physicians, physicians in training). Because we conducted all of these studies with tools we developed, there is considerable uncertainty about the applicability of our findings to other decision aids and contexts. Nonetheless, it serves as a proof-of-concept that can be applied to study the fidelity with which clinicians adhere to SDM interventions in a variety of clinical contexts and used by those developing decision aids to assess their training and implementation strategies. Albeit imperfect, this is, to our knowledge, the first assessment of the fidelity of use of point-of-care tools to support SDM.

## Conclusion

Clinicians’ fidelity to intended use of point-of-care decision aids in randomized trials was suboptimal and suggests that those trials may have underestimated the efficacy of decision aids when used as intended. Alternatively, these findings challenge our assumptions of what are key components of effective decision aids and the role of decision aids in creating meaningful conversations that facilitate SDM.

### Practice implications

The trials from which we drew the encounter videos used in this study all concluded that the tools were effective in promoting SDM, improving patient knowledge, and increasing patient involvement, at the current level of fidelity. Therefore, while fidelity to the intended use of decision aids may be important, it may not be completely necessary to achieve acceptable levels of SDM. Alternatively, other factors may be more important for facilitating high levels of SDM. However, we did observe that higher clinician fidelity was associated with important SDM outcomes, suggesting that more faithful implementation of the decision aids may lead to greater patient knowledge and increased involvement of the patient by the clinician regardless of the role other factors play. Alternative implementation strategies, including innovative approaches to training in SDM and further refinement of the decision aids may provide a means to increase SDM in this context.

Long lists of barriers and facilitators for SDM have been published [20,21]. Key barriers include patient characteristics, the clinical situation, patient preferences, and time pressures, whereas key facilitators include clinician motivation, patient characteristics, and the practicality of SDM in the clinical context [21]. Our trials have shown that many of these barriers fail to operate in the context of our decision aids, including concerns for time, lack of pertinence to the patients seen, and willingness to use (with 70% to 95% of patients and clinicians declaring interest in using the tools the next time they face a similar decision across our studies). Detailed analyses within the ‘black box’ of the clinical encounter, however, reveal how tenuous the unskilled implementation of decision aids can be and how much potential efficacy may not have been realized in our trials. While the fidelity checklist highlights several behaviors thought to facilitate SDM, including transfer of knowledge and facilitation of patient understanding of difficult concepts, it is relatively mute on other facilitators, such as clinician motivation, clinician interpersonal skills and patient characteristics. While these latter facilitators are challenging to quantify given their nature, they undoubtedly play a key role in facilitating SDM. Our analysis gave some insight into the role clinician bias can play as a barrier to SDM. Although not included in the calculation of the overall fidelity score, we observed that clinicians made recommendations to patients about the course of action to take in 46% of encounters, and 75% of these recommendations were not solicited by the patient. Although it is possible that some of these recommendations reflected a skillful synthesis of the patient’s stated values and preferences with the best available evidence, the video reviewers qualitatively noted that these recommendations usually appeared to reflect personal biases the clinician had implied earlier in the clinical consultation. Although clinician recommendations in the context of these trials may not put patients at risk for physical harm, given that there was clinical equipoise with respect to all of the included decisions, patients are harmed when they are not permitted to make decisions in keeping with their personal values and preferences. Therefore, while training interventions like those used in our decision aid trials may play a role in facilitating SDM by ensuring that certain steps are followed in the use of decision aids, SDM may yet reach its full potential through addressing more basic issues of interpersonal skills and motivation of the professional and the professional culture of SDM.

Although suboptimal, when considered in the context of the minimal training used with these decision aids, the observation that a mean fidelity score of 58% was observed in our trials should be considered a success. While higher fidelity scores appear to be associated with important outcomes and thus are optimal, we recognize that 100% fidelity may not be feasible for every clinical encounter. For instance, a clinician may be operating in a time-limited encounter and choose to focus on only the most pertinent aspects of the decision aid for the individual patient. Although imperfect implementation of our decision aids is not ideal, it is preferred to the alternative of the clinician making a paternalistic decision on behalf of the patient without consideration of their personal context. Alternatively, because a patient and clinician may have already discussed aspects of the patient’s care covered on the decision aid in previous discussions, a clinician may be able to forego certain aspects of the usage instructions included on the fidelity checklist. Our results did show a wide range of fidelity scores, suggesting that there are ‘bright spots’ where fidelity is perfect, and ‘dark spots’ where fidelity is zero. Further exploration of ‘bright spots’ and ‘dark spots’ may shed light on barriers and facilitators of optimal decision aid implementation. This analysis may reveal which aspects of training interventions should be emphasized and which are perhaps less important. Ultimately, while it is clear that the behaviors on the fidelity checklist are important for facilitating knowledge transfer and engagement of the patient, the role of other clinician behaviors in facilitating SDM remains unclear.

While there remain many definitions of shared decision making, all of which place variable emphasis on different clinician behaviors [2], we operationalize SDM in its most basic form as a conversation between the clinician and patient where the clinician brings knowledge based on the best available evidence and the patient brings his or her personal preferences, and the two are merged. We have shown that specific clinician behaviors included on the fidelity scale facilitate the one-way transfer of knowledge from clinician to patient and also serve to engage the patient in the clinical encounter, and therefore, these behaviors should be highlighted when decision aid use is demonstrated. However, our study leaves questions about other facilitators of SDM when decision aids are used, such as personal attributes of the clinician, explicit values clarification exercises, and the culture of the medical practice. It is also unclear what role patients might play in facilitating SDM, as our study only analyzed clinician behaviors. We have also only analyzed shared decision making in the context of decision aids, fully realizing that decision aids are not a prerequisite for SDM.

Our findings may justify an emphasis on the need for a patient-centered culture in which to frame SDM. While it is plausible that the unsolicited recommendations clinicians made in the context of these trials were offered according to the clinicians’ understanding of patients’ personal needs, values and preferences in accordance with SDM, our reviewers judged that the vast majority of unsolicited recommendations seemed to instead reflect the clinicians’ personal agendas. This finding, if replicated, would suggest that clinicians are still reluctant to let patients participate in decision making when the views of the patient might differ from those of the clinician [12]. Together, poor clinician fidelity to the presentation of options with their pros and cons and failure to frame the discussion within the context of uncertainty about a clear ‘best’ course of action represent poor clinician uptake and implementation of core tenets of SDM.

An alternative implication of our findings is that the elements of decision aid use we thought were key are in fact not technically necessary to promote SDM, thus explaining the lack of correlation of fidelity with other trial outcomes. As explained above, the fidelity checklist only takes into account certain quantifiable behaviors, but other factors including interpersonal skills, motivation of the professional, and the professional culture certainly play a role. This questions the validity of the checklist as a standalone tool and more importantly of our assumptions about how decision aids work. Readers can directly judge the face validity of our fidelity checklist (see Additional file 1). Also, it may be important to debate whether all checklist items should carry the same weight or whether some items should be considered essential or given greater weight.

Given our findings, we invite the community conducting research on SDM to not leave the effects of their interventions on the clinical encounter within a ‘black box.’ It is evident that a simple checklist approach may contribute to the analysis of SDM intervention implementation, but more sophisticated video coding may yield greater insights. For instance, the fidelity checklist noted the presence or absence of behaviors, but did not note their duration or the sequence in which they manifested. Other video coding techniques may support these analyses and could be applied to the same recordings analyzed here and others as they accrue as part of the routine conduct of our work [22]. If other groups were to also video record encounters – both intervention and control – we could conduct more comparative work and pool results, increasing precision and applicability.

## Competing interests

The authors of this study disclose no financial conflict of interest pertinent to this study. The Knowledge and Evaluation Research (KER) Unit at Mayo Clinic decides on topics of investigation, pursues funding, designs decision aids, conducts evaluation trials, and reports their findings. Investigators at the KER Unit, including authors of this manuscript, do not receive funding from any for-profit pharmaceutical or device manufacturer, nor do they receive any royalties or other monetary benefits, directly or indirectly, from the use of the decision aids. The KER unit makes effective decision aids available online free of charge at http://shareddecisions.mayoclinic.org.

## Authors’ contributions

KDW envisioned and developed the study, performed the bulk of the data collection, drafted and revised the manuscript. MEB provided statistical support, aided in interpretation of the results, wrote the results section, and critically revised the manuscript. RTA contributed substantially to the initial draft of the manuscript, performed data collection and revised the manuscript. LJP was instrumental in developing the study, including development of the fidelity checklists used, and provided critical revisions to the manuscript. VMM assisted in the design of the study and interpretation of the results in addition to critically revising the manuscript. EPH and HHT contributed to the development of included studies, including fidelity checklists, and critically revised the manuscript. AL oversaw the design and implementation of the study, including development of the fidelity checklists, assisted in interpretation of the results, and critically revised the manuscript. All authors read and approved the final manuscript.

## Supplementary Material

Additional file 1 Fidelity checklist items. Click here for file

Additional file 2 Comparison of encounters that were video recorded versus not. Click here for file
