# Supplementary material for: Peering into the black box: a meta-analysis of how clinicians use decision aids during clinical encounters
Source: Implement Sci. 2014 Feb 22;9:26. doi: 10.1186/1748-5908-9-26 (PMC3936841; doi:10.1186/1748-5908-9-26)
Supplement: Additional file 1 — Fidelity checklist items. [file 1748-5908-9-26-S1.doc]

**Additional file 1 – Fidelity Checklist Items**

**Items for All Studies**

- Was the Decision Aid (DA) utilized during the conversation?
- Decision aid was used:
  - Not presented to the patient
  - Presented or provided to the patient but without a discussion
  - To aid in a decision making process in the context of uncertainty regarding the best course of action
  - To aid in the decision making process but not within the context of uncertainty regarding the best course of action (i.e., with personal clinician bias favoring one or more options)
  - Cannot be determined
- Did the clinician make a recommendation?
  - No
  - Yes, at the patient's request
  - Yes, unsolicited by the patient on initial recommendation
- If the clinician made recommendation, it was first provided by the clinician:
  - Before presentation of the decision aid
  - During presentation of the decision aid
  - After presentation of the decision aid
  - Other (free text)
- Did the patient voice a clear preference prior to the decision making stage?
  - No
  - Yes
- The clinician voiced a recommendation (only answer if clinician voiced preference and clinician voiced preference):
  - Before the patient voiced a preference
  - After the patient voiced a preference
- The final decision made was to: (only answer if clinician voiced preference and clinician voiced preference)
  - Follow the patient's preference and clinician's recommendation, and these were the same
  - Follow the patient's preference which was different from the clinician's recommendation
  - Follow the clinician's recommendation which was different from the patient's preference
  - Other (free text)

**Diabetes Mellitus Medication Choice-Specific Questions**

- What decision was made in regards to diabetes medication during the visit?
  - Start a new medication
  - Continue current medication
  - Not start a new medication
  - No decision was made
  - Other (free text)
- What topics were discussed? (select all that apply)
  - Daily Routine
  - Side effects
  - Daily Testing
  - Low Blood Sugar
  - Cost
  - Blood Sugar
  - Weight Changes
  - Other (free text)
- List all side effects discussed (free text)
- Items included in Fidelity Score:

1. Does the clinician invite patient to choose the issue of greatest salience to the patient?
2. Does the clinician (or patient) indicate which of the diabetes medications the patient is currently on?
3. Does the clinician present more than one medication option as a choice to the patient?
4. Does the clinician provide assistance/direction in the use of the cards?
5. Does the clinician describe medication administration?
6. Does the clinician describe blood sugar monitoring?
7. Did the clinician describe at least one of the common side effects?
8. Does the clinician describe frequency of hypoglycemia associated with a medication(s)?
9. Does the clinician discuss weight change as a consideration for a medication?
10. Did the clinician describe the cost of the medication(s)?
11. Did the clinician force a review of all cards? (If “Yes”, subtract one point)

**Osteoporosis Choice-Specific Questions**

- What decision was made regarding bisphosphonates during the visit?
  - Start a bisphosphonate
  - Not start a bisphosphonate
  - No decision was made (or a decision was deferred)
  - Take vitamins and exercise instead of a bisphosphonate
  - Other (free text)
- What topics were discussed? (select all that apply)
  - Medication (bisphosphonates)
  - Side effects
  - Breaking bones
  - Instant fracture
  - Cost
  - Actual Risk
  - Benefits
  - How the patients risk was calculated
  - Options of care
  - Other (free text)
- List all side effects that were discussed (free text)
- Which options were discussed? (select all that apply)
  - Take a bisphosphonate
  - Do not take a bisphosphonate
  - Exercise and/or take vitamins instead of a bisphosphonate at this time
  - Discuss at a later time
  - Take no action
  - Other
- Did the clinician discuss (select all that apply)
  - - Calcium?
    - Vitamin D?
    - Exercise?
- Items included in Fidelity score:

1. Did the clinician describe the risk of breaking a bone numerically?
2. Did the clinician describe the risk as a natural frequency?
3. Did the clinician describe the time horizon for risk of breaking a bone?
4. Did the clinician describe the risk graphically?
5. Did the clinician describe the risk reduction numerically?
6. Did the clinician describe risk reduction as a natural frequency?
7. Did the clinician describe the time horizon for risk reduction?
8. Did the clinician describe the risk reduction graphically?
9. Did the clinician describe the frequency of gastrointestinal side effects associated with bisphosphonates?
10. Did the clinician describe the frequency of osteonecrosis of the jaw associated with bisphosphonates?
11. Did the clinician identify any of the following as a risk factor for the patient's likelihood of breaking a bone: age, prior fracture, parental hip fracture, alcohol consumption, smoking, corticosteroid use, secondary osteoporosis, BMI?
12. Did the clinician describe the cost of the medication(s)?

**Acute Myocardial Infarction Choice-Specific Questions**

- What decision was made regarding the bundle during the visit?
  - Start or continue all medications
  - Start or continue some of the medications
  - Not take or stop taking all medications
  - Prefer to decide at some other time
  - Other
- What topics were discussed? (select all that apply)
  - Medication
  - Side effects
  - Cost
  - Actual Risk
  - Benefits
  - How the patients risk was calculated
  - Other (free text)
- List all side effects that were discussed (free text)
- Items included in Fidelity score:
  1. Did the clinician describe the risk as a natural frequency?
  2. Did the clinician describe the time horizon for risk of having a heart attack?
  3. Did the clinician describe the risk graphically?
  4. Did the clinician describe risk reduction as a natural frequency?
  5. Did the clinician describe the time horizon for risk reduction?
  6. Did the clinician describe the risk reduction graphically?
  7. Did the clinician describe the duration of taking the medication?
  8. Did the clinician describe at least one of the common side effects?
  9. Did the clinician describe the frequency of at least one of the side effects?
  10. Did the clinician describe the cost of the medication(s)?
  11. Did the clinician discuss care, exercise or lifestyle changes?
  12. Did the clinician present the Medication Guide?

**Statin Choice-Specific Questions**

- What decision was made regarding statins during the visit?
  - Start a statin
  - Not start a statin
  - No decision was made
  - Other (free text)
- What topics were discussed? (select all that apply)
  - Medication
  - Side effects
  - Muscle aching
  - Cost
  - Actual Risk
  - Benefits
  - How the patients risk was calculated
  - Other (free text)
- List all side effects that were discussed (free text)
- Items included in Fidelity score:
  1. Did the clinician describe the risk as a natural frequency?
  2. Did the clinician describe the time horizon for risk of having a heart attack?
  3. Did the clinician describe the risk graphically?
  4. Did the clinician describe risk reduction as a natural frequency?
  5. Did the clinician describe the time horizon for risk reduction?
  6. Did the clinician describe the risk reduction graphically?
  7. Did the clinician describe the duration of taking the medication?
  8. Did the clinician describe at least one of the common side effects?
  9. Did the clinician describe the frequency of at least one of the side effects?
  10. Did the clinician describe the cost of the medication(s)?
  11. Did the clinician discuss diabetes care, exercise, or lifestyle changes?

**Chest Pain Choice-Specific Questions**

- Did study coordinator intervene or participate in the intervention?
- What decision was made during the visit?
  - To be admitted to the observation unit and have an urgent cardiac stress test
  - To follow-up with a Mayo Clinic cardiologist within 24-72 hours
  - To follow-up with patient’s primary care physician at the next available appointment
  - Other (free text)
- What topics were discussed? (select all that apply)
  - Actual Risk
  - Different tests to be done
  - Diagnosis
  - Options of care
  - How the patients risk was calculated
  - Other (free text)
- Which options were discussed? (select all that apply)
  - Be admitted to the observation unit and have an urgent cardiac stress test
  - Follow up with Mayo Clinic cardiologist
  - Follow up with primary pare provider
  - Take no action at this time
  - Have the clinician decide for patient
  - Other
- Items included in Fidelity score:

1. Did the clinician specify that the decision aid depicts the risk of a heart attack or pre-heart attack within 45 days?
2. Did the clinician specify that the decision aid depicts the risk of a heart attack or pre-heart attack?
3. Did the clinician specify a time horizon for risk (regardless of whether the time horizon was accurate)?
4. Did the clinician describe the risk as a natural frequency?
5. Did the clinician describe the risk graphically?
6. Did the clinician describe at least one of the common considerations?
