# Supplementary material for: Peering into the black box: a meta-analysis of how clinicians use decision aids during clinical encounters
Source: Implement Sci. 2014 Feb 22;9:26. doi: 10.1186/1748-5908-9-26 (PMC3936841; doi:10.1186/1748-5908-9-26)
Supplement: Additional file 2 — Comparison of encounters that were video recorded versus not. [file 1748-5908-9-26-S2.docx]

Additional file 2:

Demographic comparison of video recorded encounters versus not.

|  | VR Encounters Intervention  (N=229) | Non-VR Encounters Intervention  (N=110) | P-Value^6^ |
| --- | --- | --- | --- |
| Patient characteristics | | | |
| Female, n (%) | 131 (57) | 67 (61) | 0.08 |
| Age, mean (SD) | 60 (12) | 63 (11) | 0.35 |
| K-12 education, n (%)^1^ | 74 (33) | 40 (40) | 0.63 |
| Income < 40k/year, n(%)^2^ | 74 (35) | 33 (36) | 0.57 |
| Married, n(%)^3^ | 123 (75) | 63 (57) | 0.62 |
| Commercial insurance, n(%)^4^ | 128 (62) | 54 (56) | 0.82 |
| Clinician characteristics | N=122 | N=57 |  |
| Female, n(%) | 67 (37) | 33 (39) | 0.55 |
| Encounters per clinician,  mean, median (range) | 2.2, 1  (1, 16) | 2.1, 2.0  (1, 8) | 0.08 |
| Type^5^ | | | |
| Staff physician, n(%) | 70 (58) | 33 (58) | 0.38 |
| Physician in training, n(%) | 34 (28) | 10 (17) |  |
| Nurse or nurse practitioner, n(%) | 16 (14) | 14 (24) |  |

Legend: Video recorded VR ^1^Values missing for education (VR=7, non-VR=9); ^2^Value missing for income (VR=20, non-VR=19); ^3^Values missing for marital status (VR=66, non-VR=25); ^4^Values missing for insurance (VR=24, non-VR=14); ^5^Values missing for clinician type (VR=2); ^6^Continuous: Mean and SD represent unadjusted values, p-value comes from regression model stratified by study, Categorical: Cochran-Mantel-Haenszel test statistic , stratified by study

Comparison of outcomes for video recorded encounters versus not.

| Outcome | AMI Choice | Chest Pain Choice | DAD | Diabetes Medication Choice | Osteoporosis Choice I/II |
| --- | --- | --- | --- | --- | --- |
| DCS^1^ | 0.40 | 0.24 | 0.32 | 0.62 | 0.77 |
| DCS Informed^1^ | 0.39 | 0.72 | 0.35 | 0.55 | 0.90 |
| DCS Effective^1^ | 0.43 | 0.24 | 0.18 | 0.92 | 0.69 |
| DCS Support^1^ | ~ | 0.31 | 0.65 | 0.89 | 0.86 |
| DCS Values^1^ | ~ | 0.26 | ~ | 0.95 | 0.88 |
| DCS Certainty^1^ | ~ | **0.007** | ~ | 0.27 | 0.75 |
| Knowledge^1^ | ~ | 0.34 | 0.70 | 0.30 | **0.005** |
| Knowledge of risk estimates^2^ | **0.003** | 0.99 | 0.31 | ~ | 0.10 |
| Satisfaction^2^ | 0.49 | 0.47 | 0.77 | **0.01** | 0.63 |

^1^ Generalized linear model analysis adjusted by arm and video recording, p-value for comparison of video recording versus not. ^2^ Logistic regression adjusted by arm and video recording, p-value for comparison of video recording versus not.
